# Aerobic High-Intensity Exercise Training Improves Cardiovascular Health in Older Post-menopausal Women

**DOI:** 10.3389/fragi.2021.667519

**Published:** 2021-04-23

**Authors:** Birgitte Hoier, Line Nørregaard Olsen, Maria Leinum, Tue Smith Jørgensen, Howard Henry Carter, Ylva Hellsten, Jens Bangsbo

**Affiliations:** ^1^Department of Nutrition, Exercise and Sports, University of Copenhagen, Copenhagen, Denmark; ^2^Herlev Hospital, Copenhagen, Denmark

**Keywords:** post-menopausal, age, women, exercise training, skeletal muscle, vascular function

## Abstract

The aim of this study was to determine the effect of a period of aerobic high intensity training on central- and peripheral cardiovascular parameters in older post-menopausal women. Eleven healthy post-menopausal (>10 years after menopause) women (mean age: 64 years; BMI: 25.3 kg m^−2^) completed an 8-week period of supervised, high intensity cycle training, with sessions conducted three times per week. Before and after the training period maximal oxygen uptake, body composition, popliteal artery flow mediated dilation, exercise hyperemia, arterial blood pressure, and plasma lipids were assessed. In addition, levels of estrogen related receptor α (ERRα) and vasodilator enzymes were determined in muscle biopsy samples. Training induced an 18% increase (*P* < 0.001) in maximal oxygen uptake. Plasma High-density lipoprotein (HDL) was higher (*P* < 0.05) after than before the training period. Fat mass was reduced (4.9%; *P* < 0.01), whereas lean body mass was unaltered. Mean arterial blood pressure was unchanged (91 vs. 88 mmHg; *P* = 0.058) with training. Training did not induce a change in popliteal flow mediated dilation. Exercise hyperemia at submaximal exercise was lower (*P* < 0.01; 11 and 4.6% at 10 and 16 W, respectively) after compared to before training. Muscle ERRα (~1.7-fold; *P* < 0.01) and eNOS (~1.4-fold; *P* < 0.05) were higher after the training intervention. The current study demonstrates that, in older post-menopausal women, a period of aerobic high intensity training effectively increases maximal oxygen uptake and improves the cardiovascular health profile, without a parallel improvement in conduit artery function.

## Introduction

It is well-known that women are protected from vascular disease during the first approximate five decades of their lives, an effect which is thought to largely be due to female sex hormones and, in particular, estrogen (Bairey Merz et al., 2006; Novella et al., 2012). After the menopausal transition, when estrogen levels in the female body are diminished, the risk of cardiovascular disease is rapidly increased and, at an older age, women have a similar or even slightly higher risk of cardiovascular events as men (Lerner and Kannel, 1986; Messerli et al., 1987; Taddei et al., 1996). In addition, evidence in the literature indicates that older women present a blunted response to exercise training-induced peripheral and central cardiovascular adaptations (Parker et al., 2010).

Numerous studies have verified that exercise training has several beneficial effects on cardiovascular function, including enhanced endothelial function (DeSouza et al., 2000; Nyberg et al., 2016) and reduced arterial stiffness (Tanaka et al., 2000; Moreau et al., 2003), however, studies evaluating conduit artery endothelial function with training demonstrate that post-menopausal women may not experience the same improvements as men of similar age (DeSouza et al., 2000; Pierce et al., 2011; Santos-Parker et al., 2017). In contrast, a study assessing microvascular function has shown that women in the first years after menopause gain clear microvascular improvements with aerobic training (Nyberg et al., 2016, 2017). The reason for the discrepancy in findings in these studies on conduit artery vs. microvascular function in women is unclear, however, one possibility could be the intensity of training as, in the study on microvascular function in post-menopausal women (Nyberg et al., 2016, 2017), the training intensity was substantially higher than in the studies on conduit artery function (DeSouza et al., 2000; Pierce et al., 2011; Santos-Parker et al., 2017). Moreover, a difference in age of the women in the studies could have influenced the outcome as training may be more effective in the prevention of cardiovascular disease, if initiated at or shortly after menopause (Gliemann and Hellsten, 2019). The effect of aerobic high intensity training on conduit artery function in late post-menopausal women has not been evaluated.

One of the mechanisms potentially involved in the microvascular improvements with intense aerobic training (Nyberg et al., 2016, 2017) is related to the estrogen related receptor alpha pathway (ERRα). Muscle contraction leads to activation of the estrogen related receptors (ERRs) via activation of mitogen activated protein kinase (MAPK) and peroxisome proliferator-activated receptor γ coactivator 1α (PGC-1α), ultimately causing transcriptional activation of the estrogen response element (ERE) (Wiik et al., 2009). In contrast, estrogen binds to estrogen receptors (ERs: ERα, ERβ, and GPER) and subsequently causes activation of ERE and expression of a number of target proteins (Ciana et al., 2003). The two pathways, which both can influence NO bioavailability, may be central mechanisms in protecting the cardiovascular system (Sumi and Ignarro, 2003). We have observed that upregulation of ERRα occurs in response to training in early post-menopausal but not late pre-menopausal women, which could suggest that this pathway may be more important once estrogen is lost (Nyberg et al., 2017; Gliemann and Hellsten, 2019). In accordance, lifelong well-trained women also have a higher protein level of ERRα in muscle than sedentary age-matched women (Gliemann and Hellsten, 2019). It is thus plausible that the ERRα pathway may be more responsive early after menopause, in particular as ERRα protein appears to be downregulated with time after menopause (Gliemann and Hellsten, 2019). However, the effect of an exercise training intervention on the expression of ERRα in late post-menopausal women has not been examined.

The aim of the present study was to evaluate the efficacy of aerobic high intensity training on conduit artery endothelial function, maximal oxygen uptake and cardiovascular health parameters in late post-menopausal women. The hypothesis was that a period of aerobic high intensity training in late post-menopausal women would increase maximal oxygen uptake, but lead to a limited enhancement in conduit artery function and ERRα protein expression. To test the hypothesis, late post-menopausal women completed 8 weeks of intense aerobic cycle training. Before and after the period, maximal aerobic power, popliteal flow-mediated dilation (FMD), blood pressure, plasma lipids and skeletal muscle proteins (e.g., ERRα and eNOS) were assessed. Since training involved the legs only, FMD was assessed in the popliteal artery.

## Methods

### Study Participants

Eleven post-menopausal women, 64 ± 1.2 years of age were recruited. Average time after menopause was 14 ± 1.4 years (Table 1). Predetermined inclusion criteria included ≥10 years since menopause, a sedentary lifestyle, systolic/diastolic blood pressure ≤130/85 mmHg (normotension to non-medicated stage 1 hypertension) and BMI ≤ 30 kg m^−2^. The sedentary lifestyle was defined as being physically active <2 h per week for the last 2 years. Predetermined exclusion criteria included metabolic syndrome, chronic disease or chronic medication use, current smoking or smoking within the past 10 years and hormone treatment during the past 10 years. Metabolic syndrome was assessed as positive on 3 out of the following 4 parameters: HbA1c≥6.5 mmol L^−2^, BMI>30 kg m^−2^, TG≥1.7 mmol L^−2^ and HDL <1.29 mmol L^−2^.

**Table 1:** Subject characteristics, body composition, blood pressure, blood lipids, sex hormone levels, and maximal oxygen uptake pre and post the training intervention in post-menopausal women.

**Variable**	**Pre-training** ** (*n* = 11)**	**Post-training** ** (*n* = 11)**	***P*-value**
Age (years)	64 ± 1.2		
Time after menopause (years)	14 ± 1.4		
BMI (kg m^−2^)	25.3 ± 1.3	24.9 ± 1.2^*^	*P* = 0.012
Body fat (%)	38.7 ± 2.1	37.5 ± 2.2^*^	*P* = 0.0123
Systolic blood pressure	124 ± 4.1	118 ± 3.5	*P* = 0.062
Diastolic blood pressure	75 ± 1.2	73 ± 1.1	*P* = 0.077
Mean arterial blood pressure	91 ± 2.1	88 ± 1.8	*P* = 0.058
Triglycerides (mmol L^−1^)	1.00 ± 0.13	1.12 ± 0.11	*P* = 0.26
Total cholesterol (mmol L^−1^)	5.85 ± 0.22	6.12 ± 0.19^*^	*P* < 0.05
LDL (mmol L^−1^)	3.82 ± 0.24	3.72 ± 0.22	*P* = 0.30
HDL (mmol L^−1^)	1.88 ± 0.13	2.02 ± 0.16^**^	*P* < 0.01
HbA1c (mmol L^−1^)	6.23 ± 0.08	6.32 ± 0.09	*P* = 0.19
C-reactive protein (mg L^−1^)	1.80 ± 0.40	1.60 ± 0.48	*P* = 0.56
Estradiol (nmol L^−1^)	0.10 ± 0.01	0.12 ± 0.01	*P* = 0.11
FSH (IU L^−1^)	77.8 ± 7.7	73.4 ± 8.7	*P* = 0.16
LH (IU L^−1^)	35.2 ± 2.6	31.3 ± 2.9	*P* = 0.21
VO_2_ max (mL min^−1^)	1579 ± 65	1846 ± 64^***^	*P* < 0.001
VO_2_ max (mL min^−1^ kg b.w.^−1^)	22.8 ± 1.5	27.2 ± 1.5^***^	*P* < 0.001

*Values are presented as means ± SE (n = 11)*.

* *P < 0.05;*

** *P < 0.01;*

*** *P < 0.001 significantly different from pre*.

### Ethics

The study was approved by Ethics committee of the Capitol Region of Copenhagen (Protocol no. H-16036001) and conducted in accordance with the latest guidelines of the *Declaration of Helsinki*. All participants were informed of the experimental procedures and signed an informed consent before participation. The subjects were included after the acceptance of a medical doctor based on an initial health examination involving a health interview, resting blood pressure measurements, resting blood sample for a number of clinical variables, and resting electrocardiography.

## Experimental Design

The subjects participated in one experimental day before and after an 8-week training intervention.

### Pre-screening

The included participants underwent a test for body composition by dual-energy X-ray absorptiometry (DXA), maximal oxygen uptake test on a cycle ergometer and graded maximal test on a knee extensor ergometer. The maximal oxygen uptake test and the maximal test on the knee extensor ergometer were conducted on the day of inclusion for the purpose of habituation. A second maximal oxygen uptake test and maximal test on the knee extensor ergometer were conducted at least 48 h before the experimental day.

### Maximal Oxygen Uptake

Maximal oxygen uptake (VO_2max_) was measured (Oxycon Pro; Intramedic, Denmark) during a graded exercise test on a cycle ergometer (Monark, Sweden). The test consisted of 5 min of warm-up at 50 W followed by a graded increase in workload of 25 watts per minute until exhaustion. VO_2max_ was found by the highest observed value over a 60-s period. A plateau in VO_2_ despite increased workload, attainment of maximal heart rate and/or attainment of a respiratory exchange ratio (RER) ≥1.10 served as criteria for test validation. The VO_2max_ post measurement was obtained at least 48 h after days after the last training session.

### Single Leg Knee-Extensor Model

The subjects also conducted a graded maximal test for the right leg on a knee extensor ergometer. The test consisted of 5 min of passive warm up, followed by a workload of 12 W for the first minute and subsequently an increase of 6 W per min. until exhaustion. Peak power output (iPPO) was calculated using the power output (in watts) during the test and the duration (in seconds) at the last completed step. The incremental single leg knee-extensor post measurements were performed at least 48 h after the last training session.

### Experimental Day

Participants arrived fasting at the lab and after 30 min of supine rest blood pressure was measured three consecutive times by an automatic upper arm blood pressure monitor (M7; OMRON, Vernon Hills, IL, USA) and a blood sample was drawn. Thereafter, a skeletal muscle biopsy was obtained with a Bergstrom needle after local anesthesia to the skin and fascia. Femoral arterial blood flow was determined in the right leg by ultrasound Doppler in a supine position and with the participant partially reclined in a one-leg knee-extensor ergometer. Blood flow was then determined after every 5 min during 25 min of active knee extension work conducted at 10 W for the first 15 min. and at 16 W for the last 10 min. The participants were then again placed in bed in a supine position. After blood flow was determined as back to base-line (~20–30 min), flow mediated dilation (FMD) was assessed after 5 min of occlusion in the popliteal artery of the non-exercised (left) leg (see below for description).

### Training

The participants completed 8 weeks of training with three supervised training sessions per week. Each session lasted ~60 min and consisted of stationary aerobic high intensity interval cycling on spinning cycles. The training sessions were monitored with polar heart rate monitors (Polar Electro Oy, Kempele, Finland) and designed to reach a heart rate of >80% of maximum 70% of the time and a heart rate of >90 at 10% of the time (Table 2). All training sessions were supervised by members of the research group and conducted at a training facility on the University campus.

**Table 2:** Heart rate response during the aerobic high intensity training in post-menopausal women.

**Training intensity intervals**	** <61%**	**61–70%**	**71–80%**	**81–85%**	**86–90%**	**91–95%**	**96–100%**
% Of training time spent in intensity intervals	1.8 ± 0.3	10.0 ± 0.1	27.0 ± 1.9	24.4 ± 1.8	22.8 ± 1.7	12.4 ± 1.6	2.8 ± 0.5

*The table shows training data for the intervention period (n = 11), including mean of time in the different heart rate zones, expressed as % of maximal heart rate, for the training sessions. The values are presented as means ± SE*.

### Femoral Arterial Blood Flow Determination by Ultrasound Doppler

Femoral arterial blood flow was measured using ultrasound Doppler (Vivid E9; GE Healthcare, Pittsburgh, USA) equipped with a linear probe (L9) operating at an imaging frequency of 4/8 MHz and a Doppler frequency of 4.2 MHz. The site of measurement in the common femoral artery was distal to the inguinal ligament but above the bifurcation into the superficial and profound femoral branch to avoid turbulence from the bifurcation. All recordings were obtained at the lowest possible insonation angle and always below 60°. Sample volume was maximized by choosing the widest section of the vessel and recordings were made without interference of the vessel wall. A low-velocity filter (<1.8 m s^−1^) rejected noise caused by turbulence at vascular wall. Doppler traces and B-mode images were recorded continuously and averaged over ~30 s. Arterial diameter was assessed during systole from B-mode images for each Doppler recording. Blood pressure was monitored throughout.

### Flow Mediated Dilation

The test was standardized according to guidelines (Corretti et al., 2002) and conducted by a highly experienced researcher. FMD% was calculated as the percentwise change from baseline diameter to the maximally measured diameter after reactive hyperemia. FMD image files were analyzed in a blinded manner using the independently validated software Brachial Analyzer for Research (Version 6.8.7, Medical Imaging Applications, Iowa, USA). Analysis of brachial diameters and velocity traces were performed using automated edge detection, and used in the calculation of the following variables: FMD%: (max diameter - baseline diameter) ÷ baseline diameter) · 100. Blood flow (ml/min): π r^2^ · average blood velocity (cm/s) · 60. Shear rate: 4· [blood mean velocity (cm/s) ÷ Diameter (cm)].

### Muscle Biopsies

Skeletal muscle biopsies were obtained from m. v. lateralis using a Bergstrom needle with suction after local anesthesia (lidocaine, 20 mg ml^−1^, Astra Zeneca, Copenhagen, Denmark) of the skin and fascia and then immediately frozen in liquid nitrogen and stored at −80°C until analysis of protein content.

### Blood Samples

Blood samples (10 ml) were obtained from the antecubital vein at rest before and after the training period. The samples were analyzed for sex hormones and for a number of specific factors related to health.

### Calculation of Cardiovascular Risk

The 10-year risk of heart disease or stroke was estimated using the American College of Cardiology/American Heart Association (ACC/AHA) Atherosclerotic cardiovascular disease (ASCVD) algorithm published in 2013 (Goff et al., 2014), taking into account the age, sex, total cholesterol, high-density lipoprotein cholesterol (HDL), systolic and diastolic blood pressure (incl. treated or untreated status), status of diabetes mellitus and the current smoking status.

## Analysis

### Western Blot

Biopsies were freeze-dried and dissected free from fat, connective tissue and blood. Muscle tissue samples were homogenized in buffer containing 10% glycerol, 20 mM sodium-pyrophosphate, 150 mM NaCl, 50 mM HEPES, 1% Nonidet P-40, 20 mM β-glycerophosphate and proteolytic inhibitors. The samples were homogenized by a tissuelyser (Qiagen Tissuelyser II, Retsch, Haan, Germany). After rotation end-over-end for 1 h, the samples were centrifuged for 30 min at 17.500 rcf at 4°C and the lysates were collected as the supernatant. Protein concentrations were determined in the lysates using BSA standards (Pierce Reagents, Rockford, USA). Subsequently, the lysates were diluted to appropriate protein concentrations in a concentrated sample buffer (0.5 M Tris-base, DTT, SDS, glycerol and bromphenol blue). Equal amounts of total protein were loaded for each sample in wells on precasted TGX stain-free gels (4–15%) (Bio-Rad, Hercules, USA). Equal loading of protein was verified by total protein determination on the stain free gels (TGX). All samples were loaded in duplicates and samples from the same subject were always loaded on the same gel. Values presented are means of the duplicates. After gel electrophoresis, proteins were transferred (semidry) to a polyvinylidene difluoride membrane (Immobilon Transfer Membrane, Millipore, Massachusetts, USA), which was incubated with a primary antibody to: eNOS (5589, Abcam), PGI2 synthase (23668, Abcam), COX1 (53766, Abcam) or ERRα (76228, Abcam) over-night at 4°C. Membranes were washed for 5 min in Tris-buffered saline-Tween (TBST) before incubation with secondary horseradish peroxidase conjugated antibody (Dako, Glostrup, Denmark) for 1 h. After washing three times for 5 min in TBST, the membrane staining was visualized by incubation with a chemiluminescent horseradish peroxidase substrate (Luminata Forte, Merck Millipore, Darmstadt, Germany) and the images were digitalized on a ChemiDoc MP system (Bio-Rad, Hercules, USA). All proteins were expressed in arbitrary units normalized to the average of all samples loaded on the same gel.

## Statistical Analysis

Statistical analyses were performed with R (R Foundation for Statistical Computing, Vienna, Austria) using the interface RStudio (1.2.1335; RStudio Team, Boston, USA). Graphs were made in GraphPad Prism (GraphPad Software for Mac, Version 8.1.2., San Diego, CA, USA). Data are reported as mean ± SE and the significance level was set at *P* < 0.05. Sample size was determined for the primary response variables (FMD and blood flow) using R, for a pre-post design with paired data. When calculating sample size, the desired power was set at >80% and the error rate (α) at 5%. The sample size calculation revealed inclusion of 12 subjects to detect within-group differences in FMD after aerobic exercise training (Pierce et al., 2011). The sample size calculation for femoral arterial blood flow revealed inclusion of eight subjects to detect differences within the group pre and post 12 weeks of high-intensity exercise training (Nyberg et al., 2016).

Differences in baseline characteristics, body composition, oxygen uptake and performance measures, flow mediated dilation measures and skeletal muscle vascular proteins were assessed with a Student's *t*-test for paired data when comparing pre and post training intervention. A linear mixed model approach was used to detect differences in femoral artery blood flow with the intervention. For the linear mixed model, a Tukey *post-hoc* analysis was used. Subjects were specified as a repeated factor and identifier of random variation. Q-Q plots confirmed normal distribution. Pair-wise differences were analyzed by a *Post-hoc* procedure, performed with multi comparison. Non-adjusted *p*-values are reported.

A number of data points were missing for the measurements of FMD (*n* = 4/11) due to cuff malfunction (*n* = 2) and varicose veins (*n* = 2). One data point was missing for the muscle protein analysis (*n* = 1) due to insufficient biopsy material. The number of subjects included in each analysis is shown in the figure legends.

## Results

### Training

Training compliance was 98% (23.5 ± 0.5 sessions) and average time spent per training session was 55:44 ± 00:28 min:s.

### Body Composition

After the training intervention, body mass (70.8 ± 3.8 vs. 69.6 ± 3.8 kg; *P* < 0.01), fat mass (27.1 ± 2.8 vs. 25.8 ± 2.8 kg; *P* < 0.01), fat percent (38.7 ± 2.1 vs. 37.5 ± 2.2%; *P* < 0.05), android fat percent (42.1 ± 3.8 vs. 40.4 ± 4.1%; *P* < 0.05), gynoid fat percent (41.0 ± 1.7 vs. 39.5 ± 1.7%; *P* < 0.01), leg fat mass (8.4 ± 0.6 vs. 8.0 ± 0.6 kg; *P* < 0.05) were lower compared to before the training intervention in the late post-menopausal women. There were no changes in lean body mass (43.5 ± 1.3 vs. 43.6 ± 1.4 kg; *P* = 0.55), body muscle mass (41.3 ± 1.3 vs. 41.4 ± 1.3 kg; *P* = 0.56), visceral fat (936 ± 196 vs. 871 ± 198 g; *P* = 0.19), leg muscle mass (13.8 ± 0.6 vs. 13.9 ± 0.6 kg; *P* = 0.17), and bone mineral density (1.1 ± 0.02 vs. 1.1 ± 0.02 g cm^−2^; *P* = 0.14) with the training intervention.

### Oxygen Uptake and Performance

Maximal oxygen uptake increased by 18% (*P* < 0.001) from 1.57 to 1.85 L min^−1^ with the training intervention (Table 1). In addition, peak power when maximal oxygen was reached was 42% higher after compared to before the training intervention (108 ± 6 W vs. 153 ± 6 W; *P* < 0.001). Peak power output (iPPO) measured in the single leg knee extensor model was unchanged with the training intervention (47.5 ± 2.7 vs. 51.1 ± 2.0; *P* = 0.12). Data on VO_2max_ included here are from eleven out of twelve subjects from the original cohort (Olsen et al., 2020). One of the subjects did not meet the criteria for minimum training sessions conducted, which was set at 20 sessions in total.

### ACC/AHA Estimated 10-Year ASCVD Risk

The 10-year ASCVD risk (in %) was lowered 8% (*P* = 0.045) from 4.8 ± 0.6% to 4.4 ± 0.7% after compared to before the training intervention.

### Effect of Acute Exercise on Femoral Blood Flow

**Figure 1:**
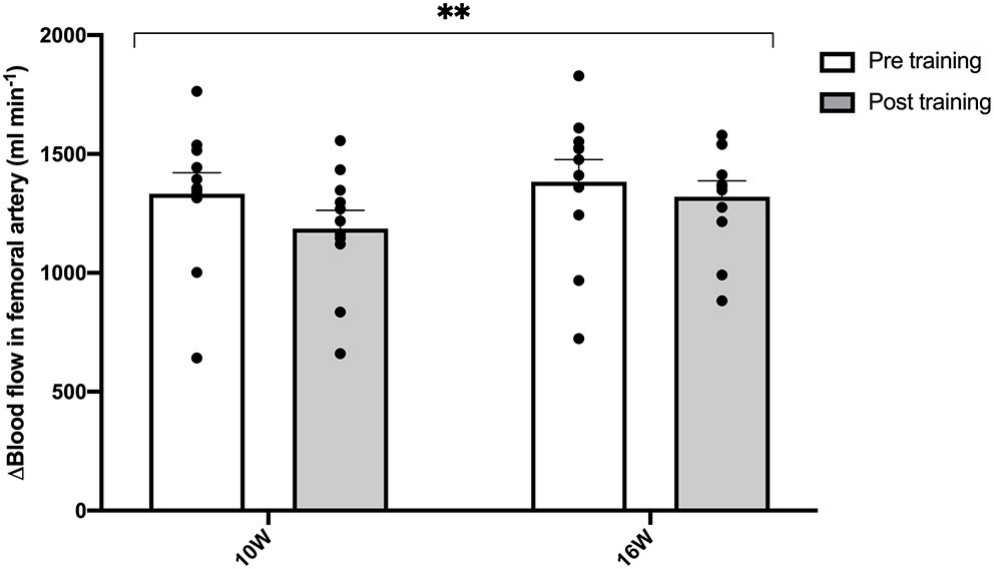
Femoral blood flow during knee extensor exercise pre and post the training intervention in post-menopausal women. Delta blood flow in femoral artery measured using ultrasound Doppler during knee extensor kicking exercise at 10 and 16W pre (open bars) and post (gray bars) training. Data are presented as means + SE and individual values (*n* = 11). **P = 0.003 significantly different from pre (main effect).

No difference was observed in femoral arterial blood flow at rest while sitting in the chair before compared to after the training period (188 ± 16 vs. 190 ± 31 ml min^−1^; *P* = 0.96). The training intervention reduced (main effect; *P* = 0.003) femoral arterial blood flow during exercise at 10 W and 16 W by 11 and 4.6%, respectively (Figure 1).

### Flow Mediated Dilation

**Figure 2:**
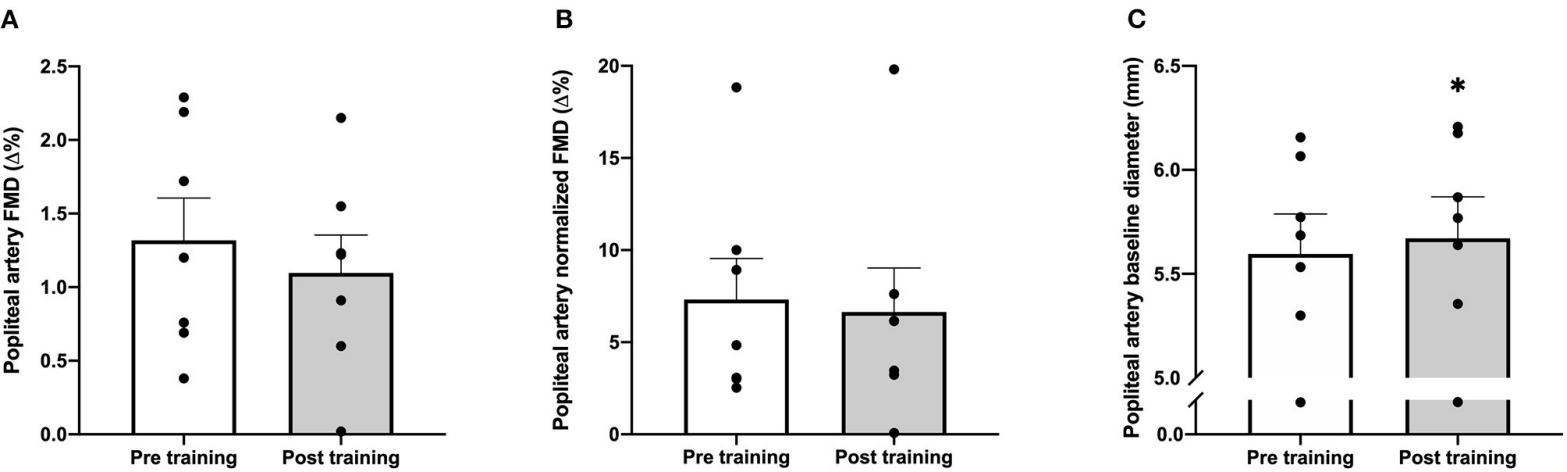
Flow mediated dilation (FMD) pre and post the training intervention in post-menopausal women. Popliteal artery FMD [%, **(A)**], popliteal artery FMD normalized to shear rate [%, **(B)**] and popliteal artery baseline diameter [mm, **(C)**] measured with ultrasound Doppler pre (open bars) and post (gray bars) 8 weeks of training. Data are presented as means **±** SE and individual values (*n* = 7). *P = 0.03 significantly different from pre training.

There was no effect of the training intervention on the popliteal artery FMD neither when measured in percentage change (*P* = 0.57; Figure 2A) or when normalized for shear rate (*P* = 0.85; Figure 2B). In addition, shear rate was not altered by the training intervention (*P* = 0.78; data not shown). Popliteal artery baseline diameter increased with the training intervention (1.3%; *P* = 0.03; Figure 2C).

### Protein Expression in Skeletal Muscle Tissue

**Figure 3:**
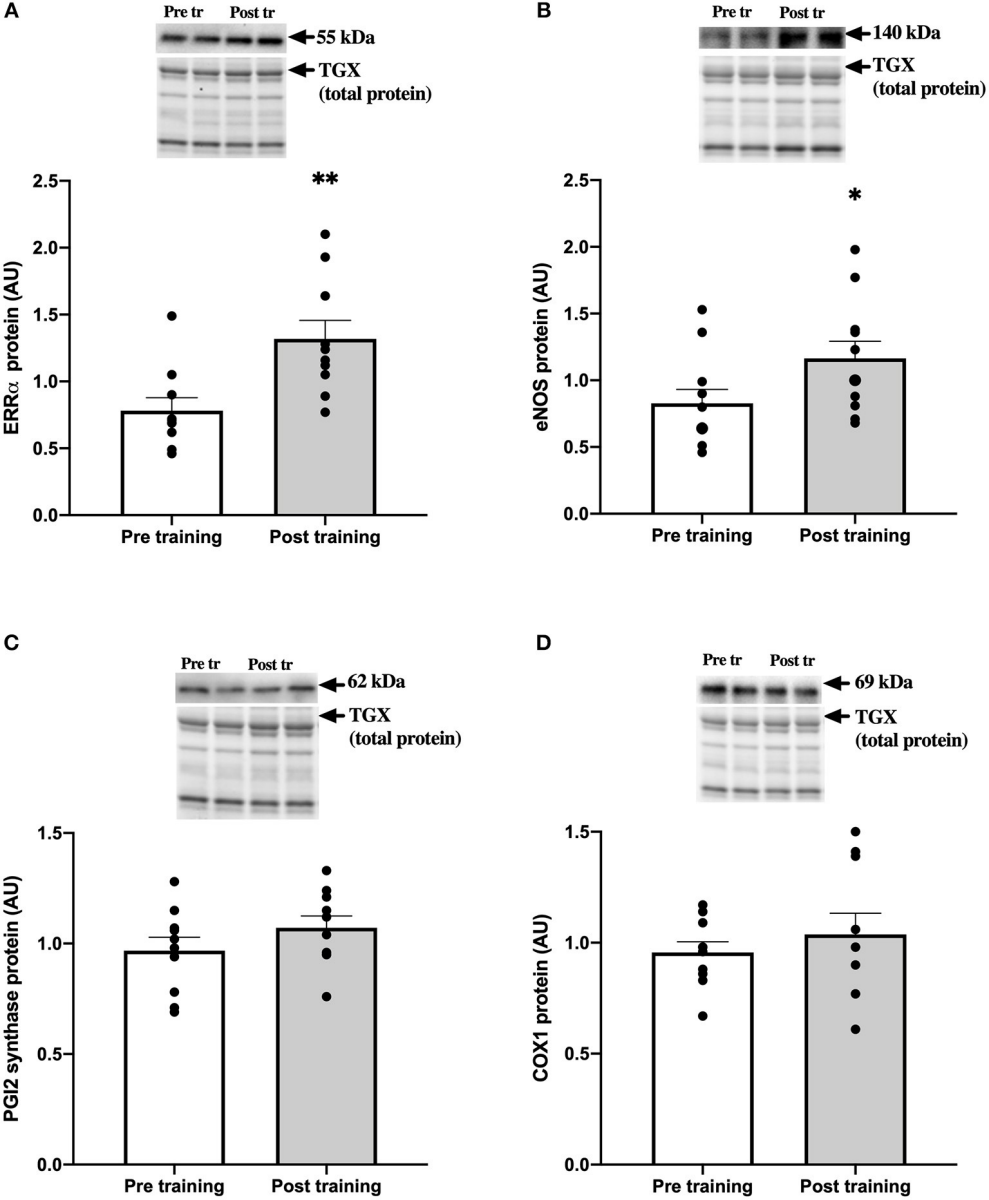
ERRα and vascular proteins in skeletal muscle pre and post the training intervention in late post-menopausal women. Resting levels of ERRα **(A)**, eNOS **(B)**, PGI2 synthase **(C)** and COX1 **(D)** protein were determined by western blot analysis on biopsies obtained from m. vastus lateralis from late post-menopausal women pre (open bars) and post (gray bars) 8 weeks of aerobic high intensity cycle training. Representative western blots for specific proteins and total protein loading control from TGX gels are presented. Data are presented as means (bars) ± SE and individual values (*n* = 10). *P = 0.042 and **P = 0.0013 significantly different from pre training levels.

There was a higher amount of ERRα (~1.7-fold; *P* = 0.0013; Figure 3A) and eNOS (~1.4-fold; *P* = 0.042; Figure 3B) in skeletal muscle tissue after compared to before the training intervention. There was no difference in the amount of muscle PGI2 synthase (*P* = 0.21; Figure 3C) or COX1 (*P* = 0.43; Figure 3D) with the training intervention.

## Discussion

The main findings of the present study were that 8 weeks of aerobic high intensity training led to a substantial increase in maximal oxygen uptake and a lowering of the overall cardiovascular risk score based on levels of BMI, fat mass, plasma HDL and blood pressure (Table 1), however, training did not affect popliteal artery flow mediated dilation.

A convincing result in the present study was the 18% increase in maximal oxygen uptake, which demonstrates that intense aerobic training effectively induces central cardiovascular adaptations in this group of women. Previous studies in post-menopausal women with more moderate intensity training interventions such as walking at 65–80% (Moreau et al., 2013) or at 70–75% (Pierce et al., 2011) of maximal heart rate have resulted in insignificant increases in maximal oxygen uptake (≤3%). The current training intervention also had a beneficial impact on cardiovascular health in that it led to a lowering of fat mass, an increase in plasma HDL and a physiologically relevant, although not statistically significant, reduction in systolic blood pressure. Based on the included measurement, the American Heart Association cardiovascular risk score was calculated for each participant before and after the training intervention and showed a significant drop from 4.8 to 4.4%. Thus, only 8 weeks of aerobic high intensity training can lower the risk of cardiovascular disease in healthy late post-menopausal women.

Despite the substantial effect of the training intervention on maximal oxygen uptake, we found no change in popliteal artery FMD with training (Figure 2). This lack of a change in FMD with training in late post-menopausal women is in agreement with studies reporting no change in brachial artery FMD after training in women past menopause (Pierce et al., 2011; Moreau et al., 2013; Santos-Parker et al., 2017). In contrast, microvascular function, as assessed by femoral arterial infusion of either acetylcholine or a prostacyclin analog, in recent post-menopausal women has been shown to be improved by intense aerobic training similar to the current study (Nyberg et al., 2016) and has also been shown to be higher in life-long trained than in sedentary older women (Gliemann et al., 2020). The current finding on absence of training induced improvements in FMD with intense aerobic training suggests that intensity of aerobic training is not a determining factor for the FMD response in this population. Instead, the finding supports the notion that conduit artery function is particularly difficult to alter in post-menopausal women with training alone and may require parallel estrogen therapy (Moreau et al., 2013). In the present study, instead of the brachial artery, the popliteal artery was used for measuring the FMD response to fit with the lower limb cycle-training intervention. A study has demonstrated a similar FMD response in brachial and popliteal artery (Parker et al., 2006). However, it is of note that the training period did induce an increase in popliteal artery baseline diameter in the present study (Figure 2C), indicating that vascular remodeling had occurred (Tinken et al., 2008).

One of the hypothesis underlying the present study was that late post-menopausal women would achieve none or only limited upregulation of ERRα and eNOS protein expression. The hypothesis was based on studies indicating a resilience to vascular adaptations with training (Pierce et al., 2011; Moreau et al., 2013; Santos-Parker et al., 2017) and the observation that the expression of ERRα appears to be diminished with time after menopause and becomes more difficult to upregulate (Gliemann and Hellsten, 2019). In contrast to our hypothesis, the 8 weeks of aerobic high intensity training did lead to higher levels of muscle ERRα and eNOS in the late post-menopausal women (Figures 3A,B), to a similar magnitude as we have observed in recent post-menopausal women (Nyberg et al., 2017). The upregulation of ERRα protein indicates that this pathway may have contributed to training induced adaptations through activation of the ERE, including upregulation of eNOS expression. However, FMD, which has been shown to be highly NO dependent (Green et al., 2014) remained unaffected by training despite the increased eNOS expression. The reason for this is unclear, however, it is possible that after the training intervention there was a change in the degree of phosphorylation of the enzyme in response to FMD.

In the present study, we found an overall decrease in femoral artery blood flow during one-legged knee-extensor exercise after the 8 weeks of aerobic high intensity training (Figure 1). Similarly, Nyberg et al. (2017) observed lower leg blood flow during submaximal knee-extensor exercise in recent post-menopausal women after a 12-week period with similar training as the current (Nyberg et al., 2017). The lowering of muscle blood flow during submaximal exercise may be explained by an enhanced potential for oxygen extraction through increased capillarization, more optimal blood flow distribution or reduced oxygen demand (Mortensen et al., 2017). As the women in this study did not present an increase in muscle capillarization with training (Olsen et al., 2020), an improved blood flow distribution or an alteration in mechanical efficiency may have been the primary reasons for the lower blood flow.

This study highlights important practical aspects. Firstly, the marked effect of aerobic high intensity training on maximal aerobic power and the overall cardiovascular risk score, including factors such as a decrease in fat mass and increase in plasma HDL (Table 1), strongly indicates that high intensity aerobic exercise is recommendable for exercise-induced cardiovascular adaptations in healthy sedentary late post-menopausal women. Secondly, it is worth noting that the aerobic high intensity cycle training modality was well-tolerated by the post-menopausal women and considering the beneficial effect on cardiovascular health, also observed in other studies (Mandrup et al., 2017), the modality is recommendable for this population.

### Study Limitations

A limitation in the present study was the relatively low number of subjects included in the study, and in particular the number of subjects for the FMD measurements. However, the individual data indicated that there was no consistent effect of training on the FMD response among the subjects, suggesting that more subjects would not have made a difference of physiological relevance. There is a possibility that the maximal oxygen uptake levels in this study were underestimated based on the criteria used (Poole and Jones, 2017). However, the participants conducted two maximal oxygen uptake tests prior to the training period, thus increasing the reliability of the determinations.

## Conclusion

In summary, intense aerobic cycle training is effective in enhancing maximal oxygen uptake and improving the cardiovascular health profile in older post-menopausal women. Moreover, the training induced increase in muscle ERRα and eNOS expression suggests that, with intense training, this pathway can be activated in late post-menopausal women. Moreover, despite the intense training regime with clear cardiovascular effects, conduit artery function, remained unaltered.

## Data Availability Statement

The data presented in this article are not readily available due to privacy or ethical restrictions. The data that support the findings of this study are available on request from the corresponding author.

## Ethics Statement

The studies involving human participants were reviewed and approved by Ethics committee of the Capitol Region of Copenhagen. The participants provided their written informed consent to participate in this study as no patients were investigated in this study.

## Author Contributions

BH, JB, and YH designed the study. BH, HC, ML, TJ, and LNO collected the data. JB, YH, BH, and LNO analyzed and interpreted the data. BH, JB, YH, and LNO drafted the article. All authors revised the article critically and approved the final version. All authors agree to be accountable for all aspects of the work. All persons designated as authors qualify for authorship.

## Conflict of Interest

The authors declare that the research was conducted in the absence of any commercial or financial relationships that could be construed as a potential conflict of interest.
